# Negotiating Closed Doors and Constraining Deadlines: The Potential of Visual Ethnography to Effectually Explore Private and Public Spaces of Motherhood and Parenting

**DOI:** 10.1177/0891241617744858

**Published:** 2017-12-14

**Authors:** Dawn Mannay, Jordon Creaghan, Dunla Gallagher, Ruby Marzella, Sherelle Mason, Melanie Morgan, Aimee Grant

**Affiliations:** 1 School of Social Sciences, Cardiff University, Cardiff, Wales, UK; 2 Hodge Lifetime, Cardiff, Wales, UK; 3 Centre for Trials Research Cardiff University, Cardiff, Wales, UK; 4 Abertawe Bro Morgannwg University Health Board, Wales, UK; 5 Teaching Personnel, Cardiff, Wales, UK

**Keywords:** visual methods, photo elicitation, motherhood, home, fieldwork

## Abstract

Pregnancy and motherhood are increasingly subjected to surveillance by medical professionals, the media, and the general public, and discourses of ideal parenting are propagated alongside an admonishment of the perceived “failing” maternal subject. However, despite this scrutiny, the mundane activities of parenting are often impervious to ethnographic forms of inquiry. Challenges for ethnographic researchers include the restrictions of becoming immersed in the private space of the home where parenting occurs and an institutional structure that discourages exploratory and long-term fieldwork. This paper draws on four studies, involving thirty-four participants, that explored their journeys into the space of parenthood and their everyday experiences. The studies all employed forms of visual ethnography, including artifacts, photo elicitation, timelines, collage, and sandboxing. The paper argues that visual methodologies can enable access to unseen aspects of parenting and engender forms of temporal extension, which can help researchers to disrupt the restrictions of tightly time bounded projects.

## Introduction

Pregnancy and early motherhood are key points where normal populations become subjected to forms of intensive medical surveillance, which have come to characterize health care (Foucault 1963). Simultaneously, within a wider sociocultural context, where the media sets the parameters of acceptable femininity, motherhood becomes a site of moral and interactional “trouble” (Lomax 2013; Mannay 2015; Skeggs 1997). Parenting increasingly occurs in the public arena in contemporary society (Boyer and Spinney 2016); however, the everyday spaces of parenthood retain their invisibility. This interplay between invisibility, periodic visibility, and stigmatizing highly visible representation has been documented in previous studies (Farough 2006; Mannay 2016).

Ethnography is interested in participating in the community, documenting patterns of life, exploring webs of meaning, and producing rich and “thick” data (Geertz 1973). This approach often involves forms of sustained observation (see Atkinson 2013; Delamont 2006; Grant 2017; Salisbury 2016; Ward 2015), but the home is often seen as a type of sanctuary, which is particularly impervious to forms of ethnographic inquiry (Lincoln 2012). The private spaces of participants’ homes are where many of the mundane practices of parenting are enacted, and although researchers may be invited into the home for interviews or other research activities, this does not constitute immersion.

For Atkinson et al. (2001, 5) a sense of social exploration and protracted investigation “gives ethnography its abiding and continuing character,” and many anthropological and ethnographic studies illustrate the benefit of prolonged forms of fieldwork, where researchers become embedded in their field of inquiry (Malinowski 1922; Willis 1977). However, protraction is threatened in an academic climate where external market contingencies create a neoliberal climate of competitiveness (Gill 2010) and continue to stress the business case for continual research output, which can erode rigorous qualitative inquiry (Mannay and Morgan 2015).

Ethnography is not simply a research technique but “an open ended, iterative, non-prescriptive vision for social science, where the researcher is encouraged to acknowledge the complexity and unpredictability of the research encounter” (Mills and Ratcliffe 2012, 155). However, the nature of short-term funding requirements often prevents researchers from indulging in “slow science,” which engenders flexibility and serendipity—as a combination of both chance and intuitive reasoning (Rivoal and Salazar 2013). These temporal restrictions raise questions about what can be achieved and whether such time-bounded work can be called ethnographic.

Where the doors to participant observation remain firmly closed and constraining deadlines impact heavily on the design and implementation of projects, then it becomes increasingly difficult for researchers to “delve into the life of the everyday of their participants” and “walk their walk, and talk their talk” (Russell and Barley 2016). Consequently, researchers need to find techniques that can still generate ethnographic understandings of their field of inquiry. Many have turned to qualitative interviewing, but this approach has been critiqued. For example, Hammersley (2006, 9) argues that work presented as ethnographic has too often “relied very heavily, or even entirely, on interviews.” More recently, Delamont (2012) has written about her despair that too much qualitative research uses interviews rather than other forms of ethnography.

However, interviews do not have to be an alternative to or incongruous with ethnography. For example, Sherman Heyl (2007) argues that the technique of interviewing can be framed as an ethnographic undertaking. Additionally, Mannay and Morgan (2015) contend that the space of the interview can be extended when researchers consider the “waiting field” and take into account the times of waiting prior to interviews, at points of interruption, and in the processes of reflexivity. Visual data production and other qualitative creative techniques can also be particularly useful when ethnographic fieldwork closes down opportunities for observation (Abrahams and Ingram 2013; Hodges 2016; Lincoln 2012; Mannay 2016) and project timeframes are contracted (Mannay et al. 2017).

Accordingly, this paper explores the potentialities of visual methods to counter the barriers of short-term, small-scale research projects and allow more nuanced insights into the private and public spaces of pregnancy and parenting. The studies involved multimodal forms of creative data, and the paper argues that these facilitated access to unseen spaces, enabled a lengthening and broadening of the data set, encouraged reflexivity and attention to familiarity, and engendered an in-depth understanding of the banal everyday elements of participants’ lives. Additionally, the open nature of the visual tasks empowered participants to raise topics outside of the research agenda, which informed the design of later funding applications and studies.

## Methods

In relation to the limitations of the timeframe of their studies, the researchers deliberately applied for multiple funding opportunities within the wider theme of parenthood, resulting in four separate but linked studies. All of the studies involved two of the authors as co-applicants, Mannay and Grant, who were joined by the five remaining authors in specific research projects. Ethical approval was provided by Cardiff University’s ethics committees in the School of Medicine (studies one and four) and the School of Social Sciences (studies two and three). In the four studies, participants were invited to contribute through the researchers’ professional and personal networks, snowballing, or via information posters in community settings.

The studies all worked with participants from deprived (Communities First) areas in south Wales, UK, which form “spatial folk devils”^1^ across the socioeconomic landscape (Mannay 2015, 19). This decision was related to historical and contemporary moral panics around working-class parents in Wales. For example, the 1904 Committee on Physical Deterioration laid the blame for the high mortality rate in Welsh infants on “the feckless and ignorance of working-class mothers in matters of nutrition and hygiene” (Beddoe 2000, 21). This claim of inadequate parenting negated the actual cause, extreme poverty and lack of ventilation, hot water, drainage, and sanitation. It also established a moral imperative to educate, civilize, and police Welsh working-class communities (Aaron et al. 1994). These dominant discourses of lack remain pervasive, and the practice and performance of motherhood continues to operate within asymmetrical gendered and classed spaces in Wales (Mannay 2015; Morgan 2015), and in the UK more widely, working-class mothers are often subject to more scrutiny and criticism (Scott and Mostyn 2003; Skeggs 1997; Tyler 2008).

Intergenerational work is also “central to the project of new motherhood,” which conceptualizes the development of maternal identities (Thomson et al. 2011, 119; West 2012). Therefore, study one^2^ was interested in intergenerational accounts of infant feeding practices. Six mothers of infants aged under thirty months and their own mothers (the grandmothers) participated in the fieldwork. Intergenerational dyad interviews (Clendon 2006) were conducted with five dyads, and one intergenerational pair preferred to be interviewed separately.

For Hurdley (2006, 717), “narratives and objects inhabit the intersection of the personal and the social,” and reflecting on this work, we asked participants to select everyday objects that they associated with infant feeding and their experiences of motherhood more generally. Artifacts were selected rather than other forms of visual data production as we did not want to ask participants to engage with time-consuming or complex pre-tasks given the pressures of new motherhood. Four mothers and three grandmothers brought a range of artifacts, including bottles, breast pumps, infant clothing, photographs, and books.

All of the elicitation interviews around these visual artifacts were conducted by Marzella, who had previously conducted a similar study (Marzella 2014). Marzella did not have any children, and this was seen as advantageous as there could be no direct comparisons between infant feeding practices, which could have acted to frame the interview discussion and position one method of feeding as more acceptable.

Study two^3^ was interested in the everyday practices of young motherhood in relation to wider mediated forms of idealized and stigmatized parenting. Mannay conducted two focus groups with three mothers in each group; mothers had given birth to their first child between the ages of sixteen and twenty-two, and four of the participants had been previously living in homeless hostels or mother and baby units. Toward the end of the focus groups, a series of ten images were introduced depicting photographs of mothers and babies that had been drawn from Google images using the terms *mother and baby* or *young mother and baby*. Photo elicitation (Collier 1957) enabled participants to explore and discuss wider conceptualizations of motherhood and reflect on the semiotics of the two sets of images, the disjuncture between the sets, and how they were framed as young mothers by general publics.

Study three^4^ extended the study two activities with a focus on the everyday practices of young parenthood. The study involved an analysis of 167 images that examined the representation of contemporary forms of motherhood in relation to social class and age, which have been reported elsewhere. Of interest here was its inclusion of further qualitative interviews, drawing on the photo-elicitation activity employed in study two. Creaghan conducted a focus group with four fathers, three aged between twenty-three and twenty-eight and one father aged thirty-nine; their partners were in their early twenties. The focus group with fathers reflected on both their own and their account of their partners’ experiences. Mason conducted individual interviews with two mothers who had their first child between the ages of sixteen and twenty-three but were “new” mothers again in their forties. The individual interviews included additional questions around the differences and similarities of being a younger and older mother.

The proposal for study four^5^ reflected the emphasis that mothers had placed on the experience of pregnancy in the earlier studies. The study worked with ten mothers who were less than thirty weeks pregnant in their initial interview, with a follow-up interview conducted before the birth. Prior to the first interview, seven of the women created a timeline that facilitated a life history interview (Adriansen 2012; Berends 2011; Mannay and Creaghan 2016). Prior to the second interview, mothers were sent a collage kit (n = 4 completed) and a word bubble activity (n = 6 completed) to represent their feelings around their pregnancy. They could engage with one, both, or none of these pre-tasks depending on their own personal preferences.

During the second interview, participants discussed the pre-activities, and nine women completed a sandboxing exercise (Lowenfeld 1979; Mannay, Staples, and Edwards 2017) to metaphorically illustrate the impact of pregnancy on their everyday lives. In contrast to study one, when a researcher who was not a mother was selected to work with participants, in the sandboxing activity, the shared experience of motherhood was used to facilitate discussion around these topics. The researchers were either pregnant, Gallagher, or already had children, Mannay and Morgan. In the sandboxing activity, both researcher and participant built a sand scene using a tray filled with sand and figures and miniatures, including animals, people, fantasy figures, everyday objects, trees, and fences. The interview could then form more of a discussion between two women about their differential understandings of this shared physiological experience.

The four studies combined generated a rich seam of data; twenty-four artifacts were brought to the interviews, and participants produced seven timelines, four collages, six word bubble activities, and nine sandboxes. There were over twenty-nine hours of recorded discussions that generated approximately 323,000 transcribed words. Data production and analysis were conducted concurrently, with emergent themes being explored in future interviews and focus groups. The visual products created by participants, which were photographed at the point of data production, acted as tools of elicitation rather than objects of analysis per se; however, they were considered in the analysis to clarify and extend the associated interview transcripts. All interview and focus group data were transcribed verbatim and analyzed applying a thematic framework, allowing codes, categories, and themes to be constructed from the data.

## What Can Visual Methodologies Enable?

Creative methods are often positioned as participatory. However, there is a danger of linking the visual with the participatory, not least because visual and narrative outputs cannot speak for themselves and they are produced through dynamic and unequal relationships (Lomax et al. 2011; Packard 2008). As Gubrium, Harper, and Otanez (2015, 21) argue, the “imagined ideal of participation and the actual practice on the ground often manifest quite differently from one another.” Therefore, in drawing on visual practices, we are not suggesting that this makes our approach participatory. However, the introduction of creative approaches did engender participatory elements and allowed more time for reflection and engagement, enabled participant-led discussions, shifted the focus of the interviews beyond the perceptions of the research teams, and broadened out the field of inquiry.

### Excavating the Artifacts

The introduction of artifacts in study one enabled an extension of time and space within the temporal constraints and fixed positioning of the interview. Participants brought the expected objects, bottles, baby formula, and breast pumps, but they also brought other items of significance such as baby books, photograph albums, or cards that generated new stories that were beyond the interview themes envisaged by the researchers (see Grant, Mannay, and Marzella 2017). For example, one mother and grandmother pair brought some coffee mugs that represented the mother’s experience of pregnancy.

As Wenham (2015) argues, pregnancy, particularly for young parents residing in marginalized areas, can be characterized by vulnerability, uncertainty, and a “fragile” self-identity, which can be compounded by forms of external regulation, and the mugs were a concrete illustration of regulation. The participants described how a waiter acted “like the kinda food police” and refused to serve them the afternoon tea they had been expecting. The waiter had listed what could and could not be eaten by pregnant women and would not allow any of the food he prescribed as off limits to be served to their table. The interview schedule had been geared toward intergenerational views and experiences of infant feeding, but the introduction of artifacts allowed participants to shift this temporal focus from the postnatal to the antenatal, where they felt surveillance activities and judgment first began.

Accordingly, the artifacts acted as tools of elicitation, leading the conversations and enabling participants to introduce what they felt was worth talking about rather than fixing them within the defined structure of set interview questions. The artifacts metaphorically took the researchers to different times and places, but they also physically shifted the interview setting from the confines of one room. Ethnographic study with the private space of the home is contentious (Lincoln 2012), but as participants introduced artifacts, there was an opportunity to become more embedded in the geography of mothering.

Dodman (2003) has also illustrated how the camera allows insights to unseen elements, and as participants led an artifact tour, we were introduced to baby boxes and wardrobes that were located in areas beyond the space selected for the interview. These tours illustrated the materiality of new purchases and hand-me-downs and how the limited space in participants’ relatively small homes had been adapted to make room for another family member. The artifacts also enabled reflections on mothers’ experiences in public spaces, outside of the home, as in the example of the “food police” waiter. Accordingly, these objects offered opportunities to reflect on and respond to normative and oppressive narratives of mothering that characterized the mundane activities of going out and being seen as well as opening up the private space of the home.

Infant feeding is a sensitive area in which policy agendas and contemporary notions of the “good mother frame infant feeding practices, rendering them a site of moral and interactional trouble” (Lomax 2013, 97). As discussed earlier, Marzella, a researcher without any children, conducted the interviews. It was anticipated that the absence of any direct experience of infant feeding would help to negate implied or assumed representations of the “right” feeding practice. Additionally, participants generally led the first part of the interview through their objects, providing an immediate narrative of their infant feeding practices. Opportunities for participants to direct the interview conversations were reduced when they had not brought any artifacts. These interviews were shorter and more focused on feeding practices with less attention to the wider ambiguities of mothering.

In one interview, the initial framing of a participant had a negative impact. In this case, unlike all of the other interviews, the mother and grandmother had chosen to be interviewed separately. They did not bring any artifacts, and the dynamic of the conversation shifted from one led by the participant to one led by the researcher. As the first interviewees had begun by talking about their objects, Marzella had easily been able to negotiate rapport and had been lulled into a “false sense of security.” Even the most efficient researcher can encounter unforeseen problems (Kvale 1996), and in this case, an early slip in positioning the mother as breastfeeding when she was formula feeding had consequences for the whole interview.

The interview only lasted for sixteen minutes, which was far shorter than all of the dyad interviews, even when accounting for two speakers. The researcher was already aware of the moral maze of infant feeding and the associated judgments (Deane 2014; Grant 2016; Hoddinott et al. 2012) and spent the interview trying to justify formula feeding as equal to breastfeeding. The interview became focused on creating a narrative where formula feeding needed to be rationalized and the researcher tried to maintain and create an acceptable form of motherhood for the participant. Consequently, the interview became a “mirror” of the researcher’s discomfort rather than a “doorway” to understanding the experience of the participant (Schwalbe 1996).

Artifacts, then, can open up the spaces and topics of interviews, allow fresh insights, and move beyond the prescriptive format of the interview guide (Mannay 2016). They can enable participants to take the lead in interviews, help to build a rapport, and set the tone and framing for the research relationship. However, participants cannot always be expected to engage with additional activities, and the researcher should not assume that they will. Here, a lesson was learned about assuming that the interviews would follow the same pattern and being unprepared for building rapport, beginning the conversation with a more general chat, and treading carefully around the sensitive topic of infant feeding.

### Photo Elicitation in Focus

As Coffey (1999) contends, fieldwork shapes and constructs identities, intimate relations, an emotional self, and a physical self, and photo elicitation, as discussed later in this section, was introduced in part to attend to the embodied aspects of distance, nearness, familiarity, and relational positioning. In studies two and three, rather than privileging a lack of connection in the researchers’ experience, we were interested in engendering some form of affinity. The notion of being an insider or an outsider is inadequate in an absolute sense, and complex and multifaceted experiences of researchers means that they are “neither total ‘insiders’ nor ‘outsiders’ in relation to the individuals they interview” (Song and Parker 1995, 243). However, working with parents, who were often young and residing in marginalized areas, and well aware of the associated stigma (Mannay 2015; Skeggs 1997; Tyler 2008), necessitated researchers who could be seen in some aspects as “like them.”

Mannay had both resided in a similar marginalized area and been a young parent. Creaghan and Mason were not parents; however, they were “researcher near” in that they were the children of teenage parents and were linked to the participants in terms of social class and geography. Arguably, this positioning engendered a sense of familiarity and to some extent created a “safe space” for discussions. The mothers and fathers were also drawn from existing friendship groups and local networks, which further enabled a comfortable space for discussion.

The interviews took place with mothers and fathers but also some of their children, reflecting the difficulties of securing child care for single parents and parents whose partners work. The focus groups were fast-moving with continual talk, and in some cases, the movement and play of infants engendered a more naturalistic session that resonated with their everyday lives. The conversations focused on the everyday activities of parenthood, interactions with family and service providers, and also encounters with general publics. As in study one, the concepts of judgment and surveillance were a common theme and a feeling that despite being a parent and knowing their child’s needs, they were often dismissed or ignored, and this was linked with both their age and residing in a marginalized area. For example, there were numerous accounts of interactions with health professionals, landlords, and other service providers;

*It does my head in cos they always . . . you do find like there’s always people that speak down to you like you’re a little kid even though you’re a parent like . . . or it’s like at the doctors when there’s something wrong and you’re like there’s something wrong with my child and you’re telling me there’s not . . . and they don’t listen because you’re young.*

Mothers and fathers who had been in mother and baby units or other forms of supported temporary accommodation also reflected on the tensions in these private yet public spaces. They described how the staff spoke to them like children, shouted at them, and used forms of surveillance such as cameras in shared living spaces and baby monitors:

*Constantly on my back watching what I’m doing watching how I’m doing it . . . nothing nothing’s private.*

These processes were framed by staff as guidance and support for young parents, but participants interpreted them as obtrusive and unhelpful. They described how they were expected to respond immediately to their children and how leaving them in cots sleeping while they prepared food, cleaned, or went outside to smoke was vilified. This was framed as unrealistic as when they moved to their own house, particularly as single parents, they would need to leave their children unaccompanied for short periods in a cot or baby seat to peg washing on the line, complete other domestic tasks, or smoke.

*Well it’s like if you like live in a house and you wanna go out the back to smoke a fag . . . you’re gonna leave the kid in the living room.*

The focus groups and interviews elicited a nuanced data set and were an effective method of generating talk and an insight into participants’ experiences, where the mundane became extraordinary in relation to external surveillance, power relations, and deleterious expectations. However, it was important to move away from the contained space of the participants’ home and friendship groups and find a way to bring “the outside in” to create “spaces of interruption and disruption” (Mannay and Morgan 2015). The focus group talk was often focused on “the other,” outsiders and their negative views around young parents, and it was essential to further explore these “commonsense” understandings that shape and are shaped by individuals’ meaning-making.

The media plays a crucial role in the circulation of ideas and the development of new social representations (Morant 1998), and through repetition across different media, specific figures accrete form and accrue affective value in ways that have significant social and political impact (Tyler 2008). Therefore, photo elicitation was included to explore how images of motherhood are interpreted, accepted, and rejected by participants. The images discussed came from the search engine Google images using the terms *mother and baby* and *young mother and baby*.

The five photographs in each category were printed out in color on A4 paper, and they were discussed with reference to the search terms applied. The images from the *mother and baby* search, featuring happy, attractive, glowing mothers with their children, were seen as “*fake*” and communicating “*I’ve got my child . . . I’ve got a happy life I’ve got everything*.” The participants understood that images are used in advertising and do not necessarily reflect actual motherhood but also considered the ways in which they communicate idealized forms, which are untenable, noting the lack of tiredness and other features of their lived experiences. Nevertheless, they were also aware of how these images, however unrealistic, were seen as the standard of acceptable motherhood and how the images representing “young mother and baby” communicated a failed maternal subject.

The young mother and baby images were seen as “*disgusting*” by the participants, and they represented mothers smoking, being distant from their children, and with unhappy facial expressions. The picture of a young mother smoking near her baby was seen as particularly offensive; one participant commented, “*that’s typical cos we all sit on sit on a bench with our babies smoking a fag*,” and another said “*it is annoying with the smoking one because it is it’s like its assumptions init*.” Health promotion has the ability to both alter society’s accepted “normal” behaviors and stigmatize those who do not conform to the public health ideal (Graham 2012; Hacking 1986), and smoking has become a visual marker of unsuitable mothering. As mentioned earlier in this section, many parents did smoke, but they were careful to do this outside of the home, away from their children (see also Holdsworth and Robinson 2008). For participants, this strategy prioritized the health of their children, but they were still vilified for leaving their children unaccompanied.

The focus group conversations had established that mothers understood their abject positioning through their day-to-day interactions. However, the images allowed an examination of the objectification of the young mother as a collective stereotype, one that guides how they are viewed as individuals. The participants explored how negative emotions and associated moral judgments become harnessed to the mediated figure of the young mother while the decontextualized and sanitized symbol of acceptable motherhood represents a fantasy; as one participant commented, “*we’re all being made out to be bad they’ve gotta be the good guys*.”

We are living in an “ocularcentric” culture (Mitchell 2011), where images form a vital part of our everyday worlds and influence both how we see ourselves and how others see us, and the photo-elicitation activity enabled a space for participants to speak back against their positioning. The participants’ conversations illustrated how they have been subject to negative assumptions, which they sometimes challenged but at other times ignored in their everyday interactions. However, being confronted with the images elicited new conversations that moved from concrete examples to a focus on wider social representations, which are abstracted from their individual experiences yet at the same time impacted on their lived realities.

Morant (1998, 253) explored representations of motherhood in media advertisements and noted that these images “work to define what forms of femininity are socially acceptable and desirable.” More recently, there have been explorations of classed visual discourses, and the figure of chav^6^ mum circulates within a wide range of media, celebrity media, reality television, comedy programming on British television, consumer culture, print media, literature, news media, ﬁlms, and “chav hate” websites (Tyler 2008). The objectification of the chav mum, young mother, and teenage mother are often framed within images, but these are not simply benign objects. Social power relations are implicit in who is seen, how they are seen, and who is viewing, and in this way, images are never “innocent” (Rose 2001).

The emotional response to the content of images is illustrated by Barthes’s (1981) concept of “punctum” as a piercing or bruising action, and more recently, Tyler (2008) has explored how visual images can engender emotions, which can be expressed as a sickening feeling of revulsion, loathing, or nausea but also through laughter. The affective impact of visual images of motherhood was apparent in the photo-elicitation activities, and young mothers and fathers articulated how differentiation and demonization were achieved in these images.

An active interface exists between viewer and visual image, which is relational, embodied, and affective (Bal 2007), and the “otherness” experienced by participants was reinforced in these images. Introducing the “found image” (Pauwels 2011) that circulates in society in an elicitation activity provided an opportunity to explore how distinctions are created not simply in the mundane activities and relationships of everyday life between individuals but also through an underlying visual tapestry where images are inert yet at the same time powerful constructions. In this way, drawing on visual images and moving beyond individual accounts offered an opportunity to develop a new layer of understanding about how participants see themselves and how they negotiate how they are seen by others.

### Pregnancy in Visual Metaphors

Study four introduced a range of visual and creative activities that were completed in situ or prior to the individual two-stage interviews in participants’ own homes, away from the “intrusive presence of the researcher” (Mannay 2013, 136). In preparation for the initial interview, participants were asked to create timelines of their lives before they became pregnant and add emotion stickers (see Gabb and Fink 2015) to reflect how they felt at different points in their biography. Timelines are useful for facilitating a recollection and sequencing of personal events denoting the “lived through life” (Adriansen 2012; Berends 2011; Mannay and Creaghan 2016; Sheridan, Chamberlain, and Dupuis 2011), and the creation of the timelines enabled an understanding of participants’ subjective understandings of their present experience and the ways in which it was inflected by their pasts.

As Edwards (2014, 180) contends, “the body imprints its own emplaced past into its present experience,” and the timeline activity helped to understand mothers’ feelings around their own pregnancy. For example, past familial relationships, educational trajectories, health issues, and previous employment impacted on participants’ negotiation of their maternal bodies. The timeline activity extended the temporal constraints of the project as it allowed participants to construct a biographic self reflexively, in their own time, and then share this with the researcher to offer a more nuanced understanding of “the now.” In the second phase, participants also completed collages or filled in speech bubbles before the interview, describing how it feels to be pregnant, which again extended the space for reflexivity.

The sandboxing activity was introduced in the second interview, not as a pre-task but as a collaborative activity. Judgments about motherhood often create difficulties in discussing everyday behaviors that conflict with the public health ideal (Graham 2012), and working on a visual task collaboratively enabled an element of sharing experiences. It was important then that the researchers could be seen as maternal subjects; Gallagher was pregnant at the time of the study, while Morgan and Mannay had children from earlier pregnancies, which enabled a form of “conscious affiliation” (Williams 1971).

Both researcher and participant built a sand scene using a tray filled with sand, figures, and miniatures, and the collaborative nature of the task allowed researchers to introduce aspects of their own experiences of “things that can’t be spoken” in relation to alcohol consumption and other health-related behaviors. Participants engaged with the sandboxing to different degrees, but many found the activity easier to complete with the researcher working alongside them on their sand scene. The sandboxing activity was also favored by participants with barriers to literacy skills as they could use visual metaphors rather than the written accounts that were required for the speech bubble activity. Additionally, those who had been concerned about producing the “right” sort of collage and not engaged with the pre-task were able to see how the researcher approached the sandbox and have a working example in situ, which increased their confidence to engage.

The sandboxing activity also confirmed the importance of the timeline activity for understanding participants’ accounts of their pregnancy and, where they had older children, their maternal relationships. For example, in her sand scene, one participant used the figure of a lion to illustrate her need to protect her children from the outside world, and this metaphor was related to her biographic account of being bullied at school and her fears for her children’s future. Another participant found it difficult to construct a sand scene; she engaged in discussing the researcher’s sand scene but did not place any figures or objects in her own tray. However, she used the sand as a canvas to etch out a single word representing her current pregnancy and being a mother to her four-year-old and seventeen-month-old children, *complete*. This was a differential form of engagement, but it was a poignant representation of being pregnant from a participant whose biography had been characterized by school-based bullying, abusive intimate relationships, and a series of three miscarriages. In terms of educational achievement and employment, the participant had also faced barriers and disappointments, but she felt secure in her abilities to be a good mother, and this new pregnancy made her feel “complete.”

The multimodal approach taken in the study confirmed previous work that illustrates the ways in which participants have differential preferences (Johnson, Pfister, and Vindrola-Padros 2012; Mannay 2016), which necessitate a need for flexibility, where different creative activities are offered and participants can decide what to engage with, in their own way, or just to talk. It also raised issues around whether visual and narrative data should be created without the intrusive presence of the researcher or whether collaboration enables participants to engage with activities more easily. In the sandboxing activity, some participants suggested that they would have been able to complete the collage if this had been done in situ with the researcher; however, other participants found it useful to reflect on their lives in their own private time and space. Again, this suggests that more choice needs to be inbuilt in to the design of the study around the nature of data production, but researchers will also be constrained by the time factors and available resources of individual projects.

It is also important to restate that the visual methods used here, although useful, were not a panacea; they did not simply create data, as the conversations relied on the relationships between researchers and participants. The researchers were pregnant or mothers, but this did not necessarily create some form of epistemic privilege. Motherhood is not a singular category, and assuming that mothers are best suited to interview mothers silences the multifaceted nature of identities, lifestyles, and perspectives and discounts crucial differences between women (Skeggs 2004). This was illustrated when Gallagher went on maternity leave and Mannay conducted a second-stage interview. In the initial interview, the participant had stated that she had easily been able to give up smoking during her pregnancies; however, in the later interview, she felt able to share that she had and still continued to smoke occasionally.

There are different aspects of familiarity between researchers and participants, and Mannay was more closely tied to the participants in terms of class, place, and maternal biography. Researchers working on familiar territory can elicit greater understanding because cultural barriers do not have to be negotiated and participants may be more open and less likely to obscure aspects of their lives (Aguilar 1981; Atkinson, Coffey, and Delamont 2003). However, the problem of overfamiliarity can also mean that the knowledge held by the insider researcher can overshadow the accounts of participants, facilitating the need to “make the familiar strange” (Delamont and Atkinson 1995). Arguably, here the visual activities helped to address the taken-for-granted cultural competence inherent to researchers’ insider status because they enable participants to independently record their own visual impressions and interpretations without the repertoire of preconceived understandings that could frame set interview questions (see Mannay 2010).

## Discussion

Reflecting on the question, “What can visual methods enable?,” this series of studies suggests that employing creative forms of data can engender a more nuanced understanding of spaces of parenthood. The home is particularly impervious to forms of ethnographic observation (Lincoln 2012), but work with artifacts allowed the researcher access to rooms in this private space. Additionally, the interview talk constructed around individual objects, assemblages of photographs, and collections of artifacts introduced topics that were beyond the interview schedule and provided new insights into the everyday negotiation of the maternal body and infant feeding practices.

The photo-elicitation activity also proved useful in disrupting the focus from individual experience to the dominant visual tropes that circulate in society to produce acceptable and unacceptable maternal subjects. The photographs created “spaces of interruption and disruption” (Mannay and Morgan 2015) that allowed connections to be made between wider social representations of motherhood and the interactions and experiences of individual participants as well as increasing participant-researcher interaction (Harper 2002).

Introducing a suite of creative narrative and visual modes of data production, including timelines, collages, text bubbles, and sandboxing, acted to broaden and deepen the data set of a relatively small-scale study. The pre-activities and collaborative work generated accounts that were biographical, current, and future oriented, allowing a more nuanced understanding of the participants’ meaning-making and approaches to parenting. However, “the greater use of visual methods is not a panacea for all ethnography’s ills nor is it the touchstone to startling ethnographic discoveries” (Ball and Smith 2001, 313), and it is important to situate these methods within the wider frame of the accompanying ethnographic interviews.

As Reavey (2011, 5) contends “the interpretation of an image cannot always be fixed,” and it was important that our own interpretations of the visual and narrative productions did not act to frame and fix the data in a way that silenced the meaning-making of the participants. Aligning with Smart’s (2009, 303) approach, we worked with the “visual data in terms of the meanings they evoke rather than for a hidden or underlying meaning” that was drawn or depicted. Creative productions are “part of the whole picture and cannot be separated from the talk” (Eldén 2012, 76); accordingly, the interviews were not so much about an understanding of the data produced as an understanding with the data produced about the lives of the participants (Mannay 2016; Radley 2011). Interviews, then, were where the meanings of visual data were communicated to the researchers.

However, there were some issues with an overreliance on visual productions to lead these interviews, and researchers need to acknowledge that some participants will not engage with pre-interview tasks. The importance of flexibility was also explored, and providing participants with a suite of activities to engage with as well as different modes of production, individual and collaborative, can be advantageous. Importantly, the positionality of the researchers also engendered differential forms of nearness and distancing, which highlights the importance of reflexivity in the embodied experience of research relationships (Coffey 1999).

However, overall, these studies outline the potential of visual ethnography to effectually explore spaces of parenthood. In attempting to enter the private space of the home and its everyday activities, they can help in negotiating closed doors and allow some insight to these hidden worlds as well as engendering reflections on more public encounters. Furthermore, in time-bounded, short-term studies, visual approaches can work against constraining deadlines and engender “the unpredictable, the tangential and the creative” (Mills and Ratcliffe 2012, 52).

Ethnographic studies may rely “very heavily, or even entirely, on interviews” (Hammersley 2006, 9), and interviews may negate access to the depth of data enabled in other forms of ethnography, such as long-term observational studies (Delamont 2012). Nevertheless, this paper has argued that interviews do not have to adopt a simple question-and-answer format; rather, they can engage with creative practices to enrich their depth and breadth and engender serendipity. Therefore, researchers faced with the difficulties of access to private spaces and the restrictions of tightly time-bounded projects, which work against traditional ethnographic practice in public spaces, may find that visual ethnography can, at least in part, negotiate both closed doors and constraining deadlines.

## Notes

1. The term *spatial folk devil* is an extension of Stanley Cohen’s (1980) work on “moral panics” and “folk devils.” Central to all moral panics, and their associated folk devils, is that the issue’s extent and significance has been exaggerated. In the original application, the folk devil often features in discourses around delinquency, youth cultures, subcultures and style, vandalism, drugs and football hooliganism, and in later work social class. In these conceptualizations, the focus has been on the individual or the group; the use of “spatial folk devil” appreciates the ways in which moral panics are also generated about particular geographical areas and housing estates so that these places become situated as stigmatized, lacking, or dangerous spatial folk devils.

2. Study one was titled *Intergenerational Views and Experiences of Breastfeeding* and was funded by the Children and Young People’s Research Network (CYPRN).

3. Study two was titled *Negotiating Young Parenthood: A Study Exploring the Ways in Which Mediated Stereotypes of Teenage Parents Impact on Their Perceptions of Their Parenting Practices and Their Engagement with Service Providers*. It was funded by the Wales Institute of Social & Economic Research, Data & Methods (WISERD) as part of the Economic and Social Research Council (ESRC) Transformative Research Seedcorn Initiative.

4. Study three was titled *Young Parents and the Media: Exploring Visual Representations and Their Everyday Impact*s. It was funded by the Cardiff Undergraduate Research Opportunities Programme (CUROP).

5. Study four was titled *Health Behaviours in Early Motherhood: Following Up a Cohort of Women from a Qualitative Longitudinal Study Using Visual Methods*. It was funded by the Wellcome Trust, Grant Reference Number 105613/Z/14/Z.

6. *Chav* is a pejorative label used to describe a particular stereotype, often referring to those living in marginalized locales and young mothers on low-incomes and state benefits.
